# Ultimate Osmosis Engineered by the Pore Geometry and Functionalization of Carbon Nanostructures

**DOI:** 10.1038/srep10597

**Published:** 2015-06-03

**Authors:** Zhigong Song, Zhiping Xu

**Affiliations:** 1 Applied Mechanics Laboratory, Department of Engineering Mechanics, and Center for Nano and Micro Mechanics, Tsinghua University, Beijing 100084, China

## Abstract

Osmosis is the key process in establishing versatile functions of cellular systems and enabling clean-water harvesting technologies. Membranes with single-atom thickness not only hold great promises in approaching the ultimate limit of these functions, but also offer an ideal test-bed to explore the underlying physical mechanisms. In this work, we explore diffusive and osmotic transport of water and ions through carbon nanotube and porous graphene based membranes by performing molecular dynamics simulations. Our comparative study shows that the cylindrical confinement in carbon nanotubes offers much higher salt rejection at similar permeability in osmosis compared to porous graphene. Moreover, chemical functionalization of the pores modulates the membrane performance by its steric and electrostatic nature, especially at small-size pores due to the fact that the optimal transport is achieved by ordered water transport near pore edges. These findings lay the ground for the ultimate design of forward osmosis membranes with optimized performance trade-off, given the capability of nano-engineering nanostructures by their geometry and chemistry.

Recent crisis of water resource shortage, especially in developing countries, has raised immersed research interests in developing new clean water technologies^1^. Designed for water desalination and wastewater treatment applications, nanostructures such as nano-sized tubules and porous monatomic layers have been explored by conducting precise measurements in experiments and molecular-level computer simulations^2^,^3^,^4^,^5^,^6^. Using porous structures with a dimension on the order of one nanometer that is close to the size of water molecules and ions, one would expect very high salt rejection in desalination or selectivity for ion separation. Moreover, research has demonstrated unexpected high permeability because of the remarkably reduced flow resistance under nanoscale constriction^7^,^8^,^9^. Carbon nanostructures, including carbon nanotubes (CNTs) that naturally possess tubular pores^2^,^10^, graphene where holes can be implanted through chemical or irradiation treatments^4^,^11^,^12^, and graphyne webs^13^,^14^,^15^,^16^ are attractive candidates for both forward and reverse osmosis (FO and RO) applications. Outstanding resistance to the mechanical load and harsh environment, as well as the feasibility to be functionalized at the atomic scale add great value to the membrane technology^17^,^18^. Considering the relatively low osmotic pressure of a few megapascals in FO applications, two-dimensional (2D) materials such as graphene with nanosized pores is much favored due to its monatomic thickness and could be considered as the ultimate material in design^19^. In addition to their promising figures of merits for energy and environmental applications, carbon nanostructures with well-defined porous structures also offer ideal platforms to explore the atomistic details of osmotic water transport as well, which is the basis for understanding biological functions of cells. For example, one could develop biomimetic designs by following the rational principles elucidated for water and ion channels in the nature^20^,^21^.

From the viewpoint of thermodynamics, the osmotic process across a semi-permeable membrane can be formulated through the van’t Hoff equation at the low-concentration limit, i.e. Δ*P* = *k*_B_*Tc*_s_, where Δ*P* is the osmotic pressure across the semi-permeable membrane and *c*_s_ is the concentration of solute^22^. Notably, this simplified description of osmotic flux does not account for the atomistic details such as types of solutes and solvents, structures of the membrane and pores, as well as their interactions. These factors could be very critical for the osmotic performance and can be clarified by performing atomistic simulations. For example, recent work has shown that the driving force of osmotic flux could be the pulling of solvent molecules across the membrane in the wake of solute-membrane collisions^10^. In thermodynamic equilibrium, solvent molecules close to the pore and in contact with the solution experience a net force towards the solvent, which is balanced by a diffusive flux of solvent particles into the solution^22^. This hopping picture of osmosis has much in common with the classic colloidal sedimentation equilibrium where an inhomogeneous colloidal density profile is maintained by the balance of a downward flux in the gravitational field and an upward diffusive flux. These concepts elucidate the molecular-level dynamics of osmotic processes in natural and synthetic systems, and pave the way for optimal design of FO applications. However, from a material design point of view, one would ask what would be the ultimate membrane for FO applications. Due to the facts we introduce above, porous carbon nanostructures are the promising candidates for this question and thus it would be interesting to explore their optimized performance by engineering their structures and chemistry.

To this end, we perform molecular dynamics (MD) simulations for a comparative study of diffusive and osmotic transport through membranes composed of porous graphene and CNTs (Fig. 1). We identify the effects of pore size for simultaneous optimization of osmotic flux and ion rejection, or so-called the performance trade-off. The role of chemical functionalization is then investigated by modifying the atomic structures and charge distribution of the pores, which exhibits notable effect on the osmotic process. The optimization is achieved by enabling ordered water transport in nanopores with specific sizes, which makes a transition to disordered flow in larger pores, where the effects of pore geometry and pore edge functionalization become insignificant. The performance of FO applications based on these membranes is discussed in this work, which is demonstrated to outperform most of conventional membranes reported in the literature.

## Results and Discussion

### Trans-membrane diffusion of water molecules

Across a semi-permeable membrane, the osmotic flux is determined by the osmotic strength (or pressure) and the permeability of solvents across the membrane. The process of water permeation depends on pore structures in the membrane, as well as the interaction between water molecules and the pore. According to the hopping picture of osmosis introduced earlier, understanding the self-diffusion of water molecules could offer some insights into the osmotic transport and is thus discussed here. We first consider slabs with width *h* next to the semi-permeable membrane. Water molecules in the slab of solvent compartment with solvent density *ρ*_solution_ hop into the solvent compartment with solvent density *ρ*_solvent_, and experience an energy penalty *hf* at the same time. Here *f* is the outward force on the water molecules in the solution arising from the fact that the total density in the solution is higher than the solvent. *f* can be related to the osmotic pressure as Δ*P* = *f*/*a*, where *a* is the slab area per solvent particle^22^. The steady-state of osmosis can be settled by the balance between diffusive fluxes across the membrane, i.e. *ρ*_solvent_ = *ρ*_solution_exp(-*fh*/*k*_B_*T*). Consequently, the osmotic flow rate is closely tied to the rate of water diffusion.

To quantify the diffusivity of water molecules across the membrane, we carry out MD simulations with two salt water chambers interfaced by the graphene or CNT membrane. We set the concentration *c* of NaCl in the solution compartment as 5 mol/L, which is comparable with the room-temperature solubility of NaCl in water (6.14 mol/L at 25 °C)^23^. We then analyze the correlation between the pore structure and diffusive flux of water *j*_W_ in thermal equilibrium, which is obtained by counting the numbers of exchanged water molecules across the membrane in both directions. We plot the diffusive fluxes as a function of the pore size in Fig. 2. Here the definition of pore size is geometrical and thus sensitive to the detailed atomic structures for small pores. Specifically, for porous graphene sheets the pore area is defined as *A*_pore_ = *n* × *A*_c_, where *n* is the number of atoms removed from the pore region and *A*_c_ is the area per atom in crystalline graphene. While for armchair (*n*, *n*) CNTs, the pore size is defined as *A*_pore_ = π*d*^2^/4, where the diameter *d* is calculated as √3*na*/π and the lattice constant of graphene *a* is 0.246 nm. It should be remarked here that for practical consideration, a depletion length of a few angstroms from the graphene edge or CNT walls should be excluded from these definitions. Our simulation results show that the diffusive water flux in activated with pore sizes of 0.052 and 0.231 nm^2^ through the porous graphene and CNT membranes by these definitions. The amplitudes of fluxes feature peak values of 4922.472 and 1978.263 L cm^−2^ per day at *A*_pore_ = 0.627 and 1.445 nm^2^, respectively. Here we evaluate the flux by referring to the area of pores, and the values should be divided by a factor of porosity for comparison with the conventional definition using the area of membranes. The water fluxes across these two types of membranes converge at large pore size above 4 nm^2^, indicating the insensitivity of the atomic structures of pores and their interaction with the water molecules at this limit.

The peaks in the diffusive flux originate from the ordered nature of water transport through the pores. To see this effect in porous graphene, we plot radial density profiles of water molecules inside the pores, which shows that for water diffusion through very small pores, the molecule has to get through a pore with comparable size with it, which results in a considerable energy barrier. As can be seen from the density profile of oxygen atoms in the water molecules (Figs. 3a and S1, S2), we find that starting from *A*_pore_ = 0.31 nm^2^, the pore is open for the lateral motion of water molecules in the pore, so the nature of single-file transport through the center of pores is broken. At a larger pore of 0.63 nm^2^, the diffusion path close to the pore edges is favored, corresponding to the peak flux measured. This result also indicates potential strong effect by functionalizing the pore edges, which could direct the order in the water transport path, and modulate the permeability as well as selectivity. As the pore size continues to increase, more paths for the water diffusion are activated, but with less ordered structures and inefficient use of the space inside the pore. As a result, the diffusive flux is reduced. For water diffusion through CNTs, similar phenomena are characterized (Fig. 3a). For CNTs with pore size below a specific value between 0.92 and 1.44 nm^2^, ordered water chain in transport is identified in the region close to the CNT walls, which results in a maximum water flux in this range. For smaller pores, the water molecules are driven through the center of CNTs that leads to significant energy penalty, while for larger pores, the atomic structures of water flow turns to be more and more disordered, as have been discussed before in the study of forced water transport through carbon nanotubes^8^,^9^,^24^. These arguments can also be clearly demonstrated from the simulation snapshots summarized in Fig. 3b.

Comparing the simulation results for porous graphene and CNTs concludes that the peak diffusive flux across porous graphene membrane is ~2.5 times higher than that across CNT membranes. To understand this, we perform steered molecular dynamics (SMD) simulations to explore the free energy profile for the diffusion of a water molecule across the membrane. The potential of mean force (PMF) data (Fig. 4a) shows that the free energy barrier Δ*G* for diffusion across graphene with a pore size of 1.10 nm^2^ is lowered by 62.2% in comparison to the CNT with a pore size of 1.44 nm^2^. This can be explained by the change in the nature of hydrogen bond (H-bond) network between water molecules during the diffusion process. Specifically, for a water molecule diffusing across porous graphene, the average number of H-bonds for each water molecule decreases slightly from the bulk value of *n*_HB_ = 3.487 to 3.137 for the pore with size of 1.10 nm^2^, while *n*_HB_ decreases significantly to 2.751 for a CNT pore of 1.44 nm^2^ (Fig. 4b and 4c). That is to say, the H-bond network remains more intact for water transport across porous graphene compared to the CNT, because the water molecules inside the pore could form contact with other molecules in both the compartments. While for molecules transport through the CNT with a pore size comparable to the length scale of the H-bond, the H-bond network has to be reconstructed or even broken due to the one-dimensional cylindrical confinement. The analysis on the H-bond network agrees with the observation that the diffusive flux is higher through porous graphene with similar pore sizes.

### Trans-membrane diffusion of ions

The diffusion of salt ions (Na^+^ and Cl^−^) is also analyzed from the MD simulation results (Fig. 2b). The diffusion of ions is initialized at larger pore sizes compared to that for the water molecules. Similarly, peaks are identified in the relation between diffusive flux *j*_I_ and the pore size. Because in our simulations both Na^+^ and Cl^−^ diffuse and the charge neutrality is maintained, so we here focus on the flux of Na^+^ ions only without the loss of generality. From the results we can conclude that the salt flux across graphene membranes is significantly higher than that for CNT membranes with the same pore size due to the different steric effects. The values of fluxes are expected to converge at a larger pore size, indicating the insensitivity to the atomic details of membranes.

The difference in the ionic diffusivity arising from the geometry of pores can be quantitatively concluded from the free energy profile of a single ion diffusing across the membrane (Fig. 4a). In bulk solvent, the ions are usually surrounded by a solvation shell, with sizes typically of 0.60 nm for Na^+^ and 0.66 nm for Cl^−^, known as the hydrated diameter, although the sizes of naked Na^+^ and Cl^−^ ions are much smaller (their crystal diameter 0.23 and 0.39 nm, respectively)^25^. As a result, when a salt ion diffuses through nano-sized pores, the hydration shells surrounding it have to be reduced because of the spatial constriction. Specifically for porous graphene, the ion should detach from the water molecules ahead of it to approach the pore. However, the contact between the ion and a new solvation shell on the other side of the membrane could be immediately established when the ion diffuse through the pore. In contrast, for ions diffusing through the CNTs with a cylindrical nanoconfinement, their solvation shell must be reduced as they enters the CNT and the dehydrated state needs to be well maintained before they diffuse across the whole nanotube and take the exit into the solution again. As a result, the free energy cost for the ions to transport through the CNTs is much higher than that for porous graphene. We analyze the pair distribution function (RDF) between the ion and water molecules based on the MD simulation trajectories. We find that the coordination number defined as the number of water molecules under the first peak of RDF curve is 6.21 for Na^+^ and 7.51 and Cl^−^ in bulk solvent. The values are reduced to 6.06, 6.85 respectivly for ions transport across porous graphene, and more significantly to 5.46, 6.62 for ions across CNTs, which are consistent with our previous discussions. It could be inferred from Fig. 2b that the threshold pore sizes for porous graphene and CNTs are 0.313 and 0.925 nm^2^, respectively, which are close to the size of hydrated ions. Specifically, for graphene pores, the cross-section areas of the hydrated ions *S*_ion_ are 0.34 nm^2^ for Cl^−^ and 0.28 nm^2^ for Na^+^ by considering them as spherical particles. While for CNT pores, the values of *S*_ion_ are 1.323 nm^2^ for Cl^−^ and 1.204 nm^2^ for Na^+^ by taking the van der Waals radius *r*_vdW_ = 0.319 nm into account, as the size of CNT pores are defined by the positions of carbon atoms.

### Osmotic flux and salt rejection

With the understanding of water and ionic transport across porous graphene and CNTs, we now turn to discuss the osmotic transport. We calculate the osmotic water flux from a pure water compartment towards the sodium chlorite (NaCl) solution. The concentration of NaCl in the solution compartment, 5 mol/L, is high enough to drive an osmotic flow over the fluctuating flux of diffusion. The measured osmotic water fluxes are summarized in Fig. 5a, which show similar peak features as in the diffusive processes. Here we define an osmotic flux *J*_O_ as the net flux directed from the pure water chamber to the solution by subtracting the diffusive backflow. Compared to the results for diffusion in Fig. 2a, the amplitudes of peak osmotic fluxes are comparable, but the characteristic pore sizes corresponding to the peak fluxes now shift to larger values for both porous graphene and CNT because of the presence of a finite osmotic pressure. As the pore size increases, the CNT membrane outperforms porous graphene as it is more resistant to the ions, which leads to a higher osmotic strength and less mixing between osmotic water flow and the ionic transport.

The salt ions start to permeate into the pure water chamber as the pore size keeps increasing. Here we quantify the ionic flow by defining a salt rejection ratio as *r*_S_ = *n*_P_/*n*_S_, where *n*_P_ is the number of salt ions permeated through the membrane and *n*_S_ is the total number of salt ions initially solved in the solution. In our work, the simulation data within a specific time interval of 20 ns are analyzed before the osmotic strength decays significantly and the equilibrium is established between the two compartments. The final values of *c* in the simulations range from ~5 molL^−1^ for nearly impermeable pores to ~3 molL^−1^ for highly permeable pores. The results summarized in Fig. 5b show that the salt rejection of CNT membranes is much higher than porous graphene, similarly as in the situation of diffusive ion transport. The threshold pore sizes are 0.627 and 1.445 nm^2^ for porous graphene and CNT membranes, respectively. Based on these results, we could conclude that although the CNT channel is much longer than the one-atom thickness of porous graphene membrane, the osmotic strength still can drive similar osmotic flow across the whole membrane, while the ultralow flow resistance by the graphitic wall does not reduce the performance. With the same density of pores in the membrane, the CNTs are thus better candidates for FO applications compared to porous graphene. However, there are some practical issues to create well-aligned and dispersed, high-density CNT arrays in the membrane. In contrast, the porous graphene could be easily created by exotic treatment such as irradiation and chemical functionalization, and controls could be made for specific size and density of pores^11^,^12^.

### Functionalization of the nanopores

**Figure 1:**
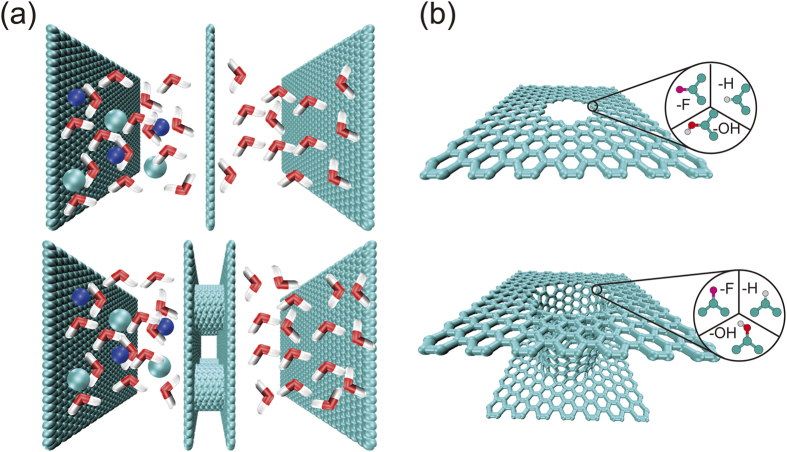
(**a**) Schematics of porous graphene and carbon nanotube membranes separating salt and pure water reservoirs. (**b**) The pore edges in graphene or at the ends of carbon nanotubes functionalized by -OH, -H and -F groups.

**Figure 2:**
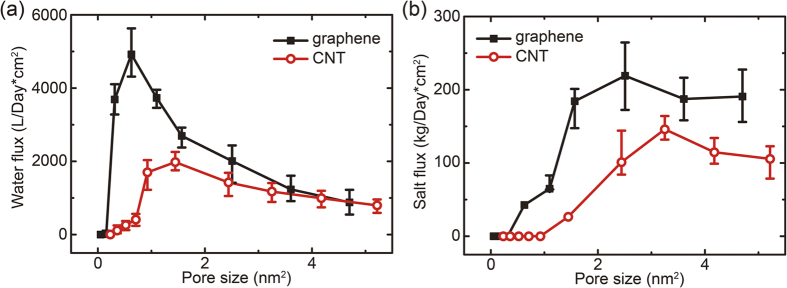
Diffusive (**a**) water and (**b**) salt ion fluxes across graphene and carbon nanotube based membranes for different pore sizes.

**Figure 3:**
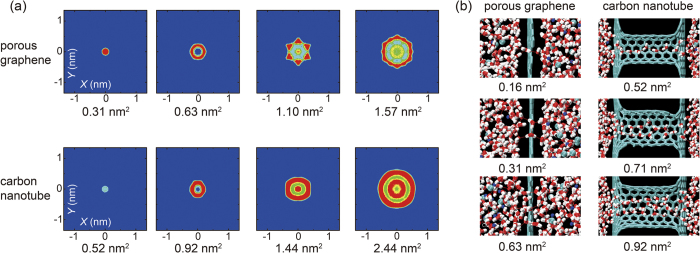
(**a**) Density distribution profiles of water molecules inside the graphene and CNT pores. The red (blue) color indicates a high (low) density. The cross section of CNTs is slightly distorted due to the formation of topological defects that covalently link them to the graphene walls. (**b**) Simulation snapshots showing the single-and multiple-file nature of water transport at small pore sizes.

**Figure 4:**
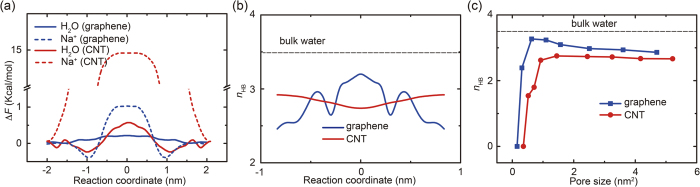
(**a**) Free energy profiles plotted as the potential of mean force for a water molecule and a sodium ion during their diffusive transport across pores in graphene with the size of 1.10 nm^2^ and carbon nanotube channels with the size of 1.44 nm^2^. The reaction coordinate is chosen as the position of oxygen or sodium atom along the pore axis, with zero at the middle of the membrane. (**b**) The average number of H-bonds per water molecule calculated during the diffusion, measured as a function of its position. (**c**) The average number of H-bonds per water molecule plotted as a function of the pore size and compared to the value in bulk water.

**Figure 5:**
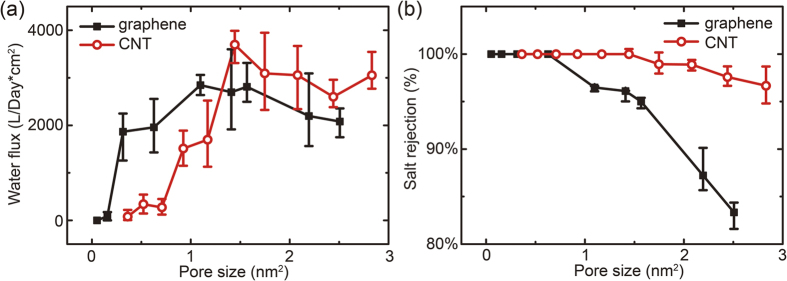
(**a**) Osmotic water flux and (**b**) salt rejection of graphene and carbon nanotube based membranes for different pore sizes.

**Figure 6:**
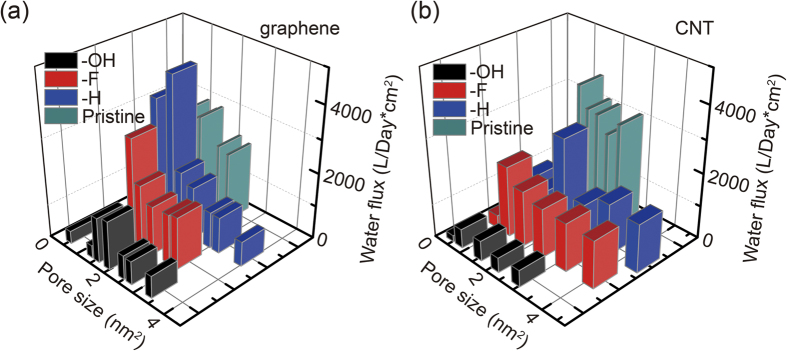
Osmotic flux through modified (**a**) graphene and (**b**) carbon nanotube membranes, with pore area up to 4 nm^2^ and chemical functionalization through edge-termination by -OH, -F, -H, respectively.

One of the additional promising features of carbon nanostructures such as graphene and CNTs is the opportunities to modify their structures at the molecular level. In addition to tailoring the pore size as we have discussed, chemical functionalization is also investigated in this study, aiming at elevating the performance of aforementioned nanoporous membranes. Here we explore the roles of chemical functionalization on the osmotic transport by considering hydrogenated, fluorinated and hydroxylized functionalization with similar sizes but different atomic charges (Fig. 1). Our MD simulation results (Fig. 6a and 6b) demonstrate notable functionalization effects for both CNT and porous graphene membranes, with strong dependence on the type of functional groups. Here the pore size is redefined by subtracting the size of functional groups. For hydrogen-terminated edges, the osmotic flux does not show significant changes compared to the pore with bare edges. The peak flux is higher through graphene pores but slightly slower in the CNT. Although charged negatively, the fluorine-termination does not change the permeability of both graphene and CNT pores as well, due to its electrostatic repulsion with the oxygen atoms in water molecules. As the bare edges of porous graphene with dangling bonds are not chemically stable in solution, and thus our findings indicate H- or F-termination provides a good protection of the edges without the loss of the performance and even higher flux at small pore sizes. In contrast, the hydroxyl group that features a similar size as F^−^ strongly prohibits the permeability because of their dipole interaction with water molecules and the formation of H-bond network. This functionalization-enabled selectivity is easy to be understood for water osmosis through pores in the single-atom-thick graphene, but not so straightforward for the CNTs as the functionalization is only available at the entrance and exit of the CNT and the whole channel in between remains intact. Moreover, we do not find notable difference in the density profile of water molecules inside the CNT channel. These facts indicate a remarkable end-functionalization effect, which could be feasibly established in experiment, as the inner walls of CNTs are difficult to be modified. We also find that the excellent salt rejection is well preserved (Figure S3), which shows very gentle dependence on the functionalization at small pore sizes.

### Assessment of FO applications

**Figure 7:**
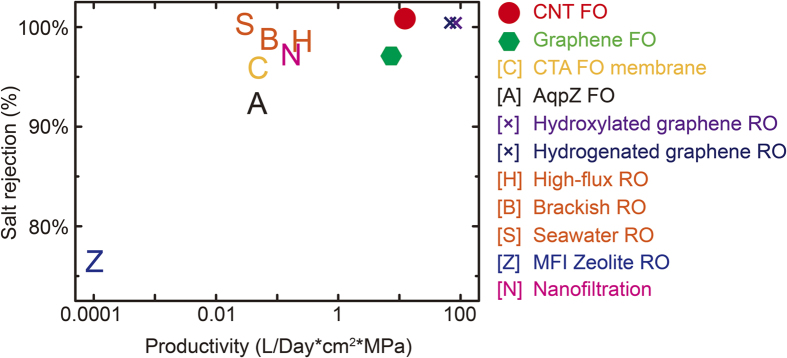
Comparison of the productivity and salt rejection of the carbon nanostructures enabled FO applications explored in this study and other technologies reported in the literature. The references can be found in the text.

Based our simulation results, the optimized pore size can be determined by balancing the osmotic flow rate and salt rejection in practical FO applications. Considering a typical setup with pore density of 10%, we compare the performance of CNT and porous graphene with various desalination techniques including both forward and reverse osmosis applications, using carbon nanostructures, aquaporin water channels, polymeric membranes^4^,^26^,^27^,^28^,^29^. The results plotted in the chart (Fig. 7) show that both nanoporous graphene and CNT based FO membranes are quite promising, which actually set a limit for related applications due to their extremely simple atomic structures and potential for functionalization based nanoengineering as addressed in this work. Practical issues such as fouling may be solved by combining electrochemical or thermal treatment of the membranes by utilizing the outstanding electrical thermal transport performance of carbon nanostructures, and structural failure can also be avoided by design the membrane architecture by efficiently utilize their outstanding mechanical resistance^17^,^30^.

In brief, we assess the performance of single-atom-thick membranes, made of embedded carbon nanotube arrays and porous graphene, in the applications of forward osmosis. We identify the peak flux that corresponds to an ordered edge-aligned water transport mechanism and the critical size to prohibit ion transport through the pores, which is much different between the CNT channel and porous graphene sheet due to a prominent steric effect. Chemical functionalization serves as a further control of the performance and hydrogen- or fluorine-termination protects the bare graphene edges for better stability in the harsh environment. The simulation results show that these membranes hold great promises in FO applications, with a balanced performance between the permeability and salt rejection that outperforms most of the conventional membranes. The simplicity of carbon nanostructures and the understandings we obtained here may also help to understand biological osmotic processes, especially the effects of atomic structures and chemical functionalization.

In the past few years, continuing efforts have been made in designing osmotic membranes using carbon nanostructures, as well as elucidating the underlying mechanisms. However, due to technical difficulties in quantifying the single-channel performance of individual carbon nanotube or porous graphene for FO and RO applications, these studies are mainly focused on the performance at the membrane level, which is made of carbon nanostructures assemblies such as their composites and porous functional graphene multilayers^31^,^32^,^33^. Only till very recently, single-channel water and ion transport have been measured in nanoscale channels^34^,^35^,^36^,^37^,^38^. Their findings suggest highly enhanced flow and ionic selectivity that validate our theoretical understandings. These progresses made substantial steps in establishing high-performance osmosis-related applications in the near future, given the developed capability in nano-engineering these nanostructures by their geometry and chemistry.

## Methods

### Atomic structures

We constructed squared 2D arrays of porous graphene and CNT membranes by using periodic boundary conditions (PBCs) with lateral dimensions of 2.982 and 2.947 nm, corresponding to a pore density of *ρ*_2D_ = 0.114 nm^−2^. Pores in graphene can be created by chemical functionalization such as oxidization or irradiation, and the pore density could reach above 0.01 nm^−2^ in experiments^11^,^12^. This choice of geometrical parameters ensures the interference between neighboring pores in the array is avoided so the osmotic dynamics can be discussed from a single-pore perspective.

Compared to the squared CNT array considered here, the density of CNTs in a membrane in a close-pack triangular lattice is defined by its diameter *d*_CNT_ measured in nm, i.e. *ρ*_2D_ = √3×(*d*+0.34)^2^/4 nm^−2^. We use the interlayer distance in graphite (0.34 nm) as the interwall distance here. In this we, we consider armchair single-walled carbon nanotubes (SWNTs) only. For a typical CNT with *d*_CNT_ = 1 nm, we have *ρ*_2D_ = 0.777 nm^−2^, about 7 times higher than the value explored here, indicating the CNT channels operate individually. To model the membranes of aligned CNTs between two liquid reservoirs, the ends of CNTs are covalently linked to a perpendicular graphene sheet through implanted topological defects as illustrated in Fig. 1. The length of CNT channel is set as 1.6 nm due to the fact that there is negligible variation of water flux by increasing this length, at least for the small-diameter CNTs explored in this work^39^.

The PBC is applied along the direction in perpendicular to the membrane (along the pore), which is sandwiched by two liquid compartments filled with water solution and solvent, respectively. Two additional plates are placed to seal these two compartments, which can move freely and are adjusted to fit the pressure difference generated during the osmosis^10^.

### Molecular dynamics (MD) simulations

We perform MD simulations in this work using the large-scale atomic/molecular massively parallel simulator (LAMMPS)^40^. The all-atom optimized potential for liquid simulations (OPLS-AA) is used for carbon nanostructures and their functional groups, which can capture essential many-body terms in inter-atomic interactions, including bond stretching, bond angle bending, van der Waals and electrostatic interactions^41^. Following previous studies on similar systems, the extended simple point charge model (SPC/E) is used for water molecules due to its predictability of dynamical properties such as the viscosity^9^,^42^,^43^,^44^. The SHAKE algorithm is applied for the stretching bond terms between oxygen and hydrogen atoms to reduce high-frequency vibrations that require shorter time steps. The interaction between water and functional groups includes both van der Waals and electrostatic terms. The former one is described by the 12-6 Lennard-Jones potential 4*ε*[(*σ*/*r*)^12^ − (*σ*/*r*)^6^] between oxygen and carbon atoms with parameters *ε*_C-O_ = 4.059 meV, *σ*_C-O_ = 0.319 nm, *ε*_C-C(-H)_ = 3.048 meV, *σ*_C-C(-H)_ = 0.355 nm, *ε*_C-C(-F)_ = 3.048 meV, *σ*_C-C(-F)_ = 0.355 nm, *ε*_F-F(-F)_ = 0.065 meV, *σ*_F-F(-F)_ = 0.384 nm, *ε*_C-C(-OH)_ = 3.048 meV, *σ*_C-C(-OH)_ = 0.355 nm, and *ε*_O-O(-OH)_ = 6.721 meV, *σ*_O-O(-OH)_ = 0.307 nm at an interatomic distance *r*^45^. In the functional groups, atomic charges are set as follows: *q*_C(-H)_ = −0.2006, *q*_H(-H)_ = 0.2006, *q*_C(-F)_ = 0.3568, *q*_F(-F)_ = −0.3568, *q*_C(-OH)_ = 0.2000, *q*_O(-OH)_ = −0.6400, *q*_H(-OH)_ = 0.4400^4^. The van der Waals forces are truncated at 1.2 nm and the long-range Coulomb interactions are computed by using the particle-particle particle-mesh (PPPM) algorithm^46^. The cations and anions are added in a way to maintain the charge neutrality. The time step of equation of motion integration is 2 fs. For temperature and pressure controls, we use the Nosé-Hoover thermostat and Berendsen barostat, respectively.

## Additional Information

**How to cite this article**: Song, Z. & Xu, Z. Ultimate Osmosis Engineered by the Pore Geometry and Functionalization of Carbon Nanostructures. *Sci. Rep*. **5**, 10597; doi: 10.1038/srep10597 (2015).

## Supplementary Material

Supplementary Information
