# Supplementary material for: Ultimate Osmosis Engineered by the Pore Geometry and Functionalization of Carbon Nanostructures
Source: Sci Rep. 2015 Jun 3;5:10597. doi: 10.1038/srep10597 (PMC4453129; doi:10.1038/srep10597)
Supplement: Supplementary Information [file srep10597-s1.pdf]

Supporting Information

Ultimate Osmosis Engineered by the Pore Geometry and Functionalization of Carbon Nanostructures

Zhigong Song and Zhiping Xu*

Applied Mechanics Laboratory, Department of Engineering Mechanics, and Center for Nano and Micro Mechanics, Tsinghua University, Beijing 100084, China

*Email: xuzp@tsinghua.edu.cn

Supplementary Figures and Captions

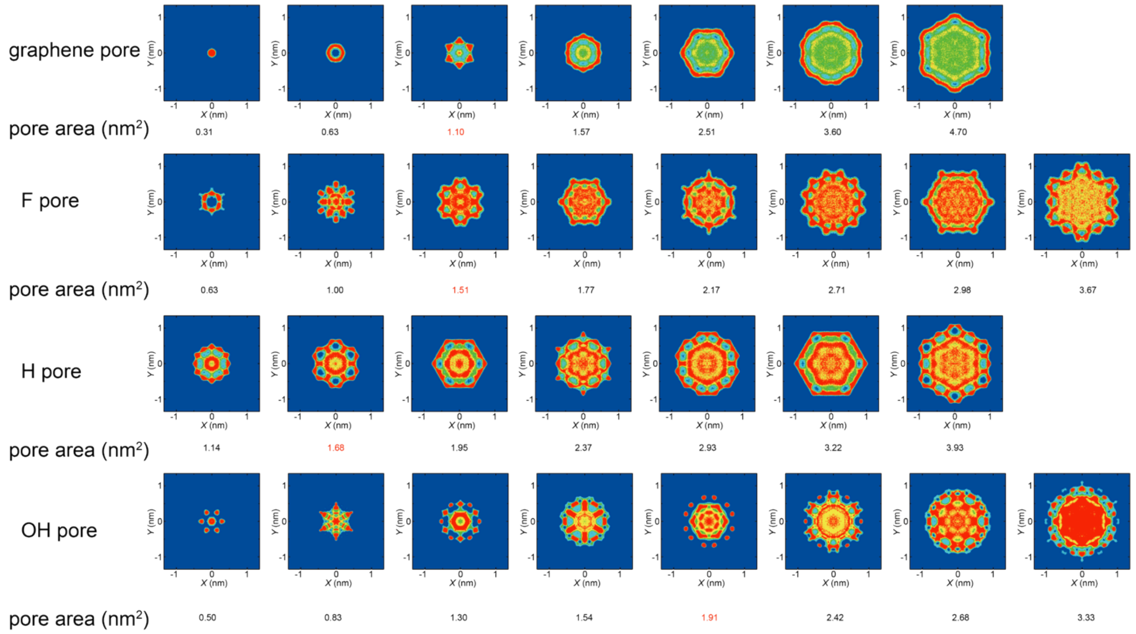

Figure S1 | Density distribution profiles of water molecules inside the pristine and functionalized pores in graphene, plotted as a function of the pore size. The red (blue) color indicates a high (low) density.

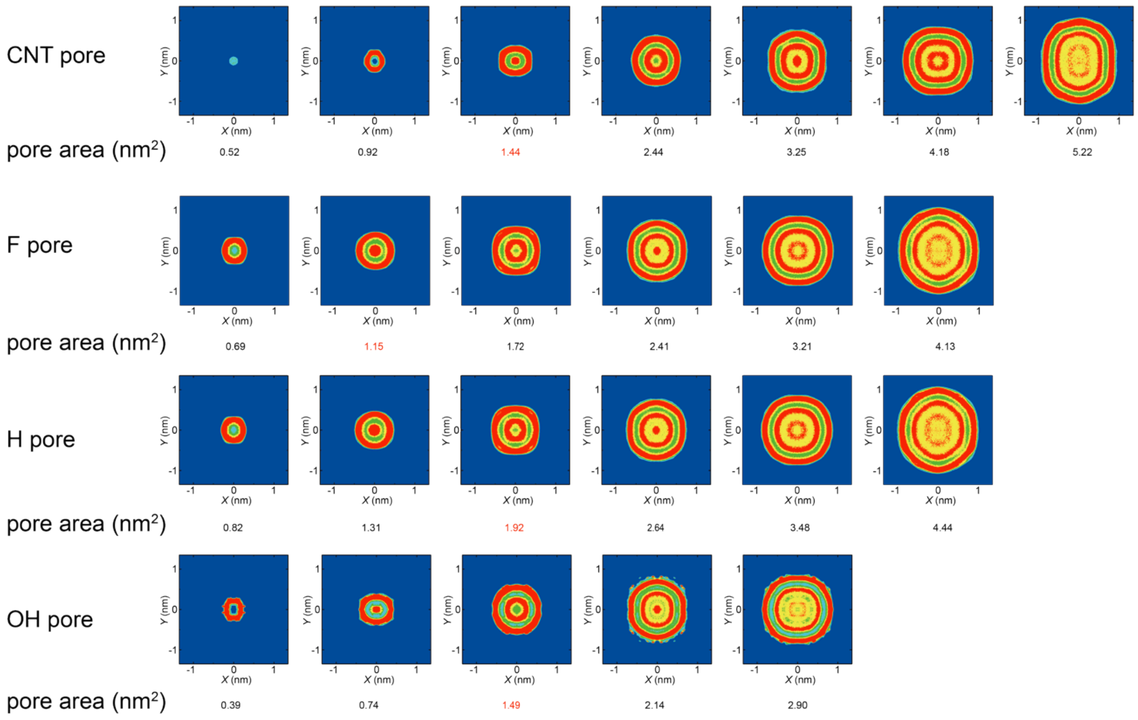

Figure S2 | Density distribution profiles of water molecules inside the pristine and functionalized carbon nanotubes, plotted as a function of the pore size. The red (blue) color indicates a high (low) density. The cross section of CNTs is slightly distorted due to the formation of topological defects that covalently linking them to the graphene walls

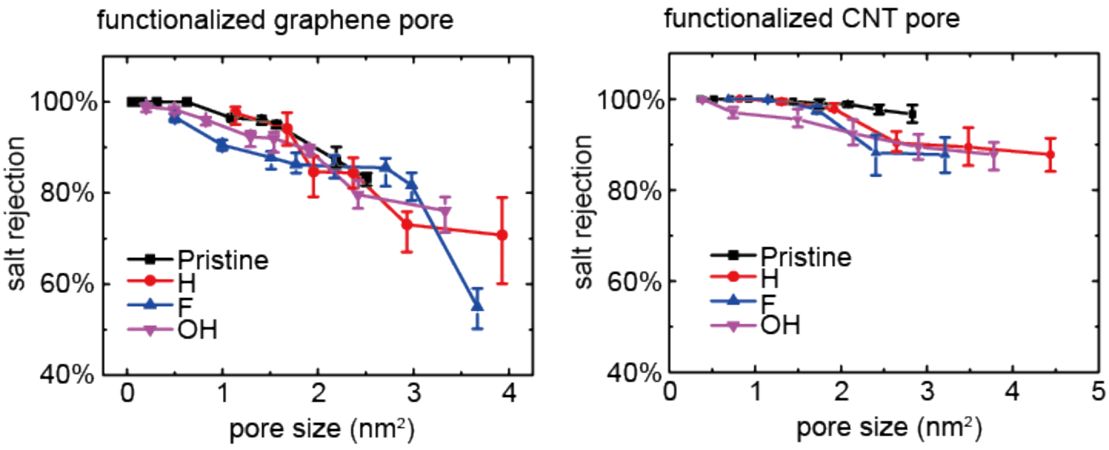

Figure S3 | Salt rejection measured for osmosis through membranes with functionalized (a) porous graphene and (b) carbon nanotube channels, plotted as a function of the pore size.
